# What Are the Optimal Sagittal Alignments in Primary Total Knee Arthroplasty: A Systematic Review and Meta‐Analysis

**DOI:** 10.1111/os.70329

**Published:** 2026-05-12

**Authors:** Guoqing Li, Yong Huang, Wang Deng, Ji Zhang

**Affiliations:** ^1^ Department of Orthopedics, National Center for Orthopaedics, Beijing Jishuitan Hospital Capital Medical University Beijing China

**Keywords:** meta‐analysis, osteoarthritis, sagittal alignments, total knee arthroplasty

## Abstract

**Objective:**

Total knee arthroplasty (TKA) is a well‐established intervention for end‐stage osteoarthritis (OA), offering substantial pain relief and functional improvement. However, a considerable proportion of patients remain dissatisfied postoperatively due to multifactorial causes. While numerous studies have investigated implant alignment, the sagittal plane alignment has received comparatively less attention, and its clinical relevance remains controversial. This systematic review aims to identify the optimal sagittal alignment (SA) parameters in TKA and to evaluate their impact on clinical outcomes.

**Methods:**

A comprehensive literature search was conducted across four databases (PubMed, the Cochrane Library, Embase, and Web of Science) from their inception to April 1, 2025. Studies focusing on SA after primary TKA were considered. Articles meeting the inclusion and exclusion criteria were subjected to meta‐analysis. The SA parameters assessed included tibial slope, posterior condylar offset, tibial and femoral component angles, femoral bowing angle, and tibiofemoral alignment.

**Results:**

The search yielded 1414 articles, after removing duplicates, of which 30 studies met the final inclusion criteria. The review confirmed that SA plays a critical role in postoperative outcomes. Malalignment in the sagittal plane was associated with complications such as instability, hyperextension, and impaired functional recovery. In particular, deviations in tibial slope and femoral bowing angle significantly influenced overall limb alignment and joint mechanics. Although achieving proper SA appears to reduce complications and improve functional outcomes, no universally accepted target values have yet been established.

**Conclusion:**

Based on current evidence, achieving optimal SA during the perioperative period is essential for improving prosthesis longevity and patient satisfaction following primary TKA. Surgeons should pay close attention to SA parameters, and the use of emerging technologies is encouraged to enhance precision in component positioning.

**Trial Registration:** PROSPERO Registration: CRD42023471336

## Graphical abstract

The optimal sagittal alignment targets for TKA remain uncertain; however, favorable sagittal alignments are hypothesized to improve prognosis and function. Discussions on sagittal alignments emphasize biomechanical, anatomical, and soft tissue‐friendly approaches to surgery.

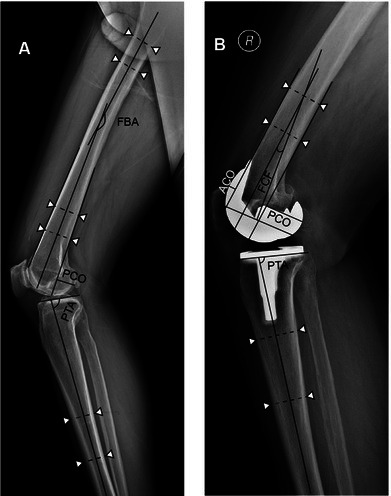

**Abbreviations**
AHRQ: Agency for Research and Health Quality
AKSS: American Knee Society score
AP: anterior–posterior
CMA: comprehensive meta‐analysis
CR: cruciate‐retaining
CT: computed tomography
DMP: distal mechanical point
DMR: distal mechanical ratio
FBA: femoral bowing angle
FCA: femoral component angle
HKA: hip–knee–ankle angle
KA: kinematic alignment
KOOS: knee injury and osteoarthritis outcome score
lPTS: lateral posterior tibial slope
MA: mechanical axis
MCID: minimal clinically important differences
MeSH: medical subject headings
mPTS: medial posterior tibial slope
NOS: Newcastle–Ottawa Scale
OA: osteoarthritis
PCO: posterior condylar offset
PROM: patient‐reported outcome measure
PS: posterior‐substituting
PSI: patient‐specific instrument
PTS: posterior tibial slope
ROM: range of motion
TCA: tibial component angle
TFA: tibial‐femoral alignment
TKA: total knee arthroplasty
TS: tibial slope

## 1 Introduction

Osteoarthritis (OA) is a highly prevalent condition and a leading cause of activity limitation in adults [1]. Total knee arthroplasty (TKA) is widely regarded as the most effective treatment for end‐stage OA, with outcomes influenced by patient characteristics, surgeon expertise, and implant alignment strategies [2, 3]. Despite advances in prosthesis design and surgical technology, approximately 20% of patients report dissatisfaction after TKA, often due to persistent pain or suboptimal functional outcomes [4, 5]. One contributing factor to patient dissatisfaction is the failure to achieve proper alignment—both in the coronal and sagittal planes—along with inaccurate prosthesis placement [6]. While most existing literature focuses on coronal alignment, SA remains insufficiently explored and continues to be a topic of debate. Given that knee joint motion predominantly occurs in the sagittal plane, accurate SA is of critical importance. Nevertheless, the precise impact of SA on clinical outcomes remains unclear, and consensus on optimal sagittal parameters has not yet been established. This systematic review aims to evaluate the role of SA in primary TKA and to identify alignment parameters that may contribute to improved clinical outcomes and patient satisfaction.

## 2 Methods

### 2.1 Protocol

A systematic search was conducted to identify studies related to SA in TKA, including aspects such as component positioning, functional outcomes, and complications. The systematic review and meta‐analysis protocol was registered with PROSPERO under registration number CRD42023471336, adhering to the guidelines outlined in the Preferred Reporting Items for Systematic Reviews and Meta‐Analyses (PRISMA) statement [7].

### 2.2 Literature Search Strategy

A comprehensive literature search was carried out across four databases: PubMed, the Cochrane Library, Embase, and Web of Science, covering publications from their inception up to April 1, 2025 (Table S1). The following medical subject headings (MeSH) terms and keywords were used: “sagittal,” “sagittal plane,” “sagittal vertical axis,” “alignment,” “balance,” “Arthroplasty, Replacement, Knee,” “Total Knee Arthroplasty,” “Knee Replacement Arthroplasty,” “Unicompartmental Knee Arthroplasty,” “Unicondylar Knee Arthroplasty,” and “Partial Knee Arthroplasty.” These terms were combined using Boolean operators. Additionally, the reference lists of all relevant articles were reviewed as per established protocols.

### 2.3 Study Selection

All retrieved records were imported into Endnote X 9.3.3. Two authors (G.L. and Y.H.) independently reviewed the titles and abstracts to identify studies relevant to the topic, excluding duplicates. Studies were included if they met the following criteria: patients undergoing primary TKA; not applicable or as reported in the original study; any clinically relevant outcomes; original studies including cohort studies and cross‐sectional studies. Studies have insufficient data to calculate effect sizes, consisting solely of abstracts, revision TKA studies, reviews, case reports, research protocols, commentaries, conference abstracts, or editorials were excluded. The full texts were then independently evaluated by G.L. and Y.H., with discrepancies resolved by consulting a third reviewer (W.D.) to reach consensus.

### 2.4 Radiological Assessment

**FIGURE 1:**
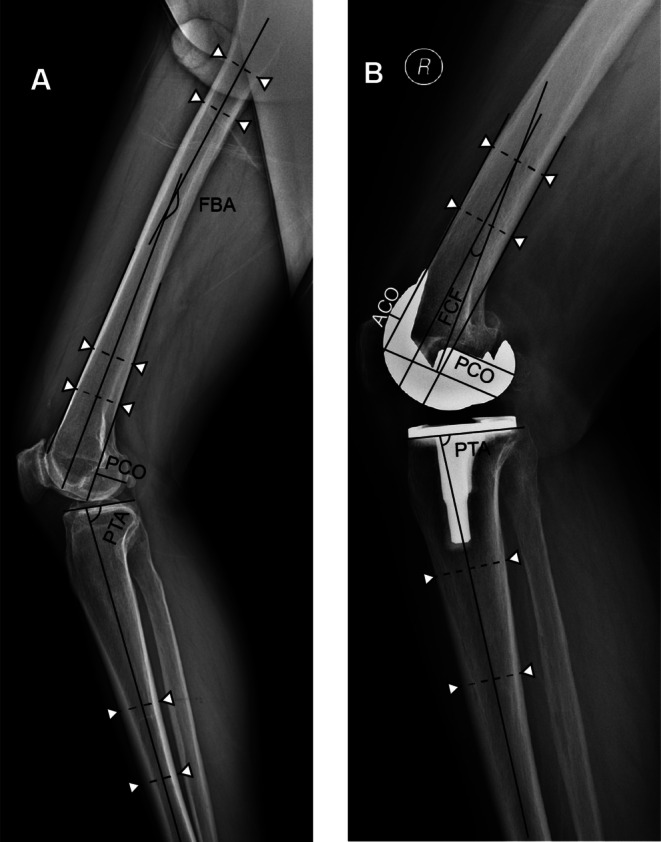
Sagittal variables in the radiological assessment. Figure 1 consists of two panels. Panel A shows a preoperative lateral knee radiograph, in which sagittal plane parameters are assessed. These include the posterior tibial slope, defined as the angle between a line connecting the anterior and posterior points on the tibial plateau and the sagittal mechanical axis; the thickness of the posterior femoral condyles, measured from a tangent line over the posterior femoral cortex; and the femoral bowing angle, defined as the angle between the distal and proximal femoral anatomical axes. Panel B displays a postoperative lateral knee radiograph, where the following sagittal alignment parameters of the knee prosthesis are evaluated: the posterior slope of the tibial component, the anterior condylar thickness, the posterior condylar thickness, and the flexion angle of the femoral component.

Plain radiographs are the primary method for evaluating TKA perioperatively, with sagittal variables measured in lateral views (Figure 1).

1. *Tibial slope (TS)*: The angle between a line connecting the anterior and posterior points on the tibial plateau and the sagittal mechanical axis (MA) (Figure 1A,B).
2. *Posterior condylar offset (PCO):* The thickness of the posterior femoral condyles, measured from a tangent line over the posterior femoral cortex (Figure 1A,B).
3. *Tibial component angle (TCA)*: The angle between the anterior tibial cortical line and the baseline of the tibial component.
4. *Femoral component angle (FCA)*: The angle between the anterior femoral cortical line and the baseline of the femoral component (Figure 1B).
5. *Femoral bowing angle (FBA)*: The angle between the distal and proximal femoral anatomical axes (Figure 1A).
6. *Tibial‐femoral alignment (TFA)*: The angle between the femoral anatomical axes and the tibial anatomical axes at maximum extension in a weight‐bearing position.

### 2.5 Quality Assessment

The quality of case–control and cohort studies was assessed using the Newcastle–Ottawa Scale (NOS), which includes three domains and eight specific criteria: selection, comparability, and outcome/exposure evaluation. Cross‐sectional studies were evaluated using the Agency for Healthcare Research and Quality (AHRQ) criteria, which consist of eleven items and are scored as “yes,” “no,” or “unclear.” Quality assessments were conducted by G.L. and Y.H., and any discrepancies were resolved by consulting a third author (W.D.).

### 2.6 Meta‐Analysis

To compare postoperative outcomes between patients with proper versus improper sagittal alignment after primary TKA, pooled ORs with 95% CIs were calculated. For interpretation, an OR < 1 indicates that improper alignment (Group B) is associated with a higher risk of adverse outcomes compared with proper alignment (Group A).

Factors influencing the posterior TS were analyzed and two groups were compared: posterior TS measured using mechanical alignment line (Group A), posterior TS measured using alternative reference line (Group B).

This meta‐analysis compared the accuracy of component alignment between two surgical techniques in primary TKA: the conventional technique and the navigated technique. The outcome of interest was the achievement of precise component alignment, treated as a dichotomous variable (accurate vs. inaccurate). An OR < 1 favored the navigated technique (i.e., higher accuracy).

To explore optimal SA in primary TKA, data extracted from the studies were analyzed. All statistical analyses were conducted using comprehensive meta‐analysis (CMA) Version 3.3.070. Statistical analysis was performed using a fixed‐effects model, which was used when the number of studies included in a meta‐analysis is less than five [8, 9]. To assess the robustness of the pooled results, we conducted sensitivity analyses by sequentially omitting one study at a time (“leave‐one‐out” method). Statistical heterogeneity was assessed using the *I* ^2^ statistic, with *I* ^2^ > 50% considered substantial heterogeneity. Publication bias was assessed using Egger's regression test, as a funnel plot was not applicable for this analysis. A two‐tailed *p* value < 0.05 was considered indicative of significant publication bias. Studies with a significant effect size or a high risk of bias were excluded. In cases where relevant data extraction or calculation of standardized effect size was not feasible, eligible studies were described narratively.

## 3 Result

### 3.1 Identification and Quality Assessment of Studies

**FIGURE 2:**
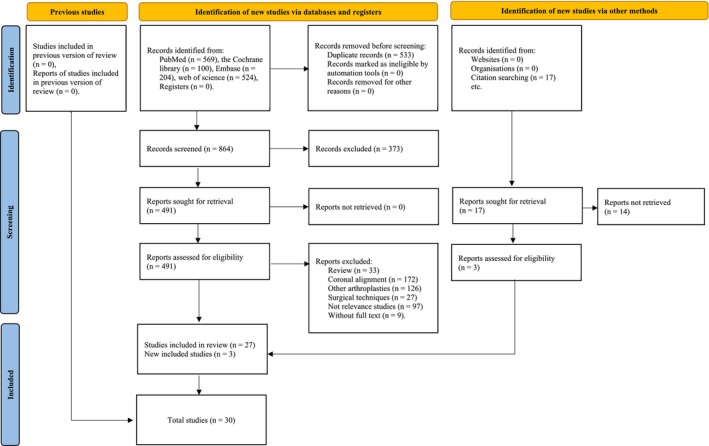
Flow diagram for systematic reviews.

The study selection process is illustrated in Figure 2. A total of 1397 articles were retrieved from PubMed (*n* = 569), the Cochrane Library (*n* = 100), Embase (*n* = 204), and Web of Science (*n* = 524). After removing 533 duplicate records, 373 records were excluded based on title screening. Subsequently, 491 full‐text articles were assessed for eligibility. Articles were excluded if they were reviews (*n* = 33), focused on coronal alignment (*n* = 172), involved other arthroplasties (*n* = 126), discussed surgical techniques (*n* = 27), were not relevant (*n* = 97), or were abstracts only (*n* = 9). Additionally, three records were added from references. Ultimately, 30 studies were included in the analysis. Of these, 21 were cohort studies, with 2 receiving 9 stars, 7 receiving 8 stars, 9 receiving 7 stars, and 3 receiving 6 stars. Eight cross‐sectional studies and one case–control study were also assessed. Further details are provided in Tables 1, 2, 3.

**TABLE 1:** Quality assessment of cohort study.

Author, country, (year)	Selection			Comparability		Exposure/outcome			Total score
	Item 1	Item 2	Item 3	Item 4	Item 5	Item 6	Item 7	Item 8	
Kaushik et al., Australia (2022)	1	1	1	1	1	1	1	NA	7
Gokhan et al., USA (2015)	1	1	1	1	1	1	NA	1	7
Jai‐Gon et al., Korea (2009)	1	1	1	NA	1	1	NA	1	6
Zhang et al., China (2021)	1	1	1	1	1	1	NA	1	7
Manjunath et al., India (2015)	1	1	1	1	1	1	1	1	8
Jobe et al., France (2022)	1	1	1	1	1	1	1	1	8
Lee et al., UK (2009)	1	1	1	1	1	1	1	1	8
David et al., UK (2020)	1	1	1	NA	1	1	1	1	7
Guruprasad et al., India (2023)	1	1	1	1	NA	1	1	1	7
Yaron et al., Israel (2022)	1	1	1	1	1	1	1	1	8
Carl et al., USA (2010)	1	1	1	NA	1	1	1	1	7
Hassan et al., USA (2023)	1	1	1	NA	1	1	NA	1	6
Hassan et al., Indiana (2021)	1	1	1	1	2	1	1	1	9
Li et al., China (2022)	1	1	1	1	2	1	1	1	9
Hiroyuki et al., Japan (2012)	1	1	1	1	1	1	NA	1	7
Chloe et al., Scotland (2019)	1	1	1	1	1	1	1	1	8
Sebastien et al., Australia (2012)	1	1	1	1	1	1	1	1	8
Takehito et al., Japan (2015)	1	1	1	1	1	1	1	1	8
Bryce et al., USA (2023)	1	1	1	1	1	1	NA	1	7
Seo et al., Korea (2018)	1	1	1	NA	1	1	NA	1	6
Solayar et al., China (2017)	1	1	1	NA	1	1	1	1	7

*Note:* For cohort studies: Selection: Item 1, representativeness of the exposed cohort; Item 2, selection of the non‐exposed cohort; Item 3, ascertainment of exposure; Item 4, demonstration that outcome of interest was not present at start of study; Comparability: Item 5, comparability of cohorts on the basis of the design or analysis; Outcome: Item 6, assessment of outcome; Item 7, was follow‐up long enough for outcomes to occur; Item 8, adequacy of follow up of cohorts.

Abbreviation: NA: not available.

**TABLE 2:** Quality assessment of cross‐sectional study.

Author, country, (year)	Item 1	Item 2	Item 3	Item 4	Item 5	Item 6	Item 7	Item 8	Item 9	Item 10	Item 11
Chen et al., China (2014)	Yes	Yes	Yes	Yes	unclear	Yes	Yes	No	Na	Yes	Na
Tadashi et al., Japan (2016)	Yes	Yes	Yes	Yes	Yes	Yes	NA	No	NA	Yes	NA
Armin et al., Germany (2016)	Yes	Yes	No	NA	Unclear	Yes	Yes	Yes	NA	Yes	NA
Hyuk et al., Korea (2008)	Yes	Yes	Yes	Yes	Unclear	Yes	Yes	Yes	NA	Yes	NA
Ryo et al., Japan (2012)	Yes	Yes	Yes	Yes	Unclear	Yes	Yes	Yes	NA	Yes	NA
Zeng et al., China (2022)	Yes	Yes	Yes	NA	Unclear	Yes	Yes	No	NA	Yes	NA
Jae et al., Korea (2008)	Yes	Yes	Yes	Yes	Yes	Yes	Yes	No	NA	Yes	NA
Douglas et al., USA (2017)	Yes	Yes	Yes	Yes	NA	Yes	Yes	Yes	NA	Yes	NA

*Note:* Item 1, Define the source of information (survey, record review); Item 2, list inclusion and exclusion criteria for exposed and unexposed subjects (cases and controls) or refer to previous publications; Item 3, indicate period used for identifying patients; Item 4, indicate whether or not subjects were consecutive if not population‐based; Item 5, indicate if evaluators of subjective components of the study were masked to other aspects of the status of the participants; Item 6, describe any assessments undertaken for quality assurance purposes; Item 7, explain any patient exclusions from analysis; Item 8, describe how confounding was assessed and/or controlled; Item 9, if applicable, explain how missing data were handled in the analysis; Item 10, summarize patient response rates and completeness of data collection; Item 11, clarify what follow‐up, if any, was expected and the percentage of patients for which incomplete data or follow‐up was obtained.

Abbreviation: NA: not available.

**TABLE 3:** Quality assessment of case–control study.

Author, country, (year)	Selection			Comparability		Exposure/outcome			
	Item 1	Item 2	Item 3	Item 4	Item 5	Item 6	Item 7	Item 8	Total score
Yoshinori et al., Japan (2019)	1	1	1	1	2	1	1	1	9

*Note:* For case–control studies: Selection: item 1, Whether case determination is appropriate; Item 2, Case representativenesst; Item 3, Selection of control; Item 4, Determination of control; Comparability: Item 5, Control major confounding factors; Item 6, Control this major confounding factor; Outcome: Item 7, Determination of exposure factors; Item 8, Determine two groups of exposure factors by the same method; Item 9, Response rate.

### 3.2 Variables in the SA

The femoral sagittal plane was defined as the plane perpendicular to the line connecting the lateral epicondylar apex and the medial epicondylar sulcus. The tibial sagittal plane was defined as the plane perpendicular to the line connecting the midpoints of the medial and lateral tibial plateaus [10]. The following variables were identified in plain radiographs: TS (*n* = 12), PCO (*n* = 2), TCA (*n* = 4), FCA (*n* = 5), FBA (*n* = 3), and TFA (*n* = 4), which were described and analyzed.

### 3.3 Vital Significance of SA

A total of seven studies examined the range of motion (ROM) in relation to SA (Table 4). Williams et al. [11] suggested that TS could influence translations, with higher slopes correlated with a greater maximum flexion angle (*p* = 0.002) and ROM (*p* = 0.031), which are crucial for achieving satisfactory in vivo implant kinematics. Shatrov et al. [12] measured TFA preoperatively (3.4° ± 5.9°) and postoperatively (0.8° ± 1.9°), concluding that external rotation of the femoral component and varus alignment of the tibial component could achieve a more balanced target. Manjunath et al. [13] observed improved knee scores (*p* = 0.0001) and functional scores (*p* = 0.0082) in patients with TFA ≤ 3°. Longstaff et al. [14] identified that optimal SA (slope 1°–5° and femoral angle −2° to 2°) was associated with a low cumulative error score, better functional outcomes (*p* = 0.015), and faster rehabilitation (*p* = 0.001).

**TABLE 4:** The significance of sagittal alignments in the TKA.

Author, country (year)	Sample (knees) methods	Results 1	Results 2	Results 3	Results 4	Findings
David et al., UK (2020)	25 (27) patients X‐ray	Higher PTS was correlated with a reduction in maximum flexion angle and flexion ROM	Higher PTS was correlated with a posterior shift in medial compartment AP translation during step‐up	Higher PTS was correlated with reduced lateral compartment AP translation during step‐down	PTS was correlated with the tibial component rotation slightly and HKA moderately	PTS influence kinematics during the step‐up and step‐down, which have implications in the debate on KA vs. MA
Jobe et al., France (2022)	102 patients Robotic system	Pre‐op vs. post‐op of TFA: 3.4° ± 5.9° vs. 0.8° ± 1.9°	KA gap balance: extension vs. flexion: 65.7% vs. 49.1%	Angle of FCP more externally rotated to PCA: KA vs. FA: 0.5° vs. 1.7°	Angle of FCP more varus to tibia: KA vs. FA: 3.0° vs. 3.5°	KA does not balance the flexion gap, with external rotated FCP and the varus TCP
Manjunath et al., India (2015)	80 (120) patients X‐ray and CT	Inliers vs. outliers of TFA: (TFA ≤ 3° vs. TFA ≥ 3°): 93.34% vs. 6.66%.	Inliers vs. outliers of TFA: KS: 83.32 ± 6.88 vs. 46.5 ± 37.47 FS: 80.35 ± 14.2 vs. 47.5 ± 38.89	sFCA inliers: 100%; sFCA: 4.15° flexion; KS vs. FS: 80.86 vs. 78.16.	sTCA inliers: 100%; sTCA: 88.4°; KS vs. FS: 80.86 vs. 78.16.	Accuracy of the alignment and position in all three planes improve better result.
Lee et al., UK (2009)	159 patients X‐ray	Pre‐ vs. postop PTS: Good: 1°–5°: fKSS: 42.1 (3.6) vs. 66.6 (5.8); tKSS 82.1 (6.1) vs. 151.6 (7.1) Bad: outside 1° to 5°; fKSS: 41.2 (5.6) vs. 65.3 (5.3); tKSS 81.3 (8.2) vs. 148.4 (7.6)	Pre‐ vs. postop sagittal femoral: Good: −2° to 2°: fKSS: 43.8 (3.8) vs. 67.1 (5.3); tKSS 85.1 (5.9) vs. 151.8 (6.5); Bad (3°, 4°): fKSS: 41.8 (6.4) vs. 68.2 (7.5); tKSS 81.6 (9.9) vs. 154 (9.7); Bad ≥ 5°; fKSS: 34.8 (8.3) vs. 58.6 (12.3); tKSS: 70.7 (14.2) vs. 139.2 (17.2)	Sagittal tibial and LOS: Good: 1°–5°: 8.8 ± 3.0 Bad: outside 1°–5°: 9.4 ± 4.3	Segittal femoral and LOS: Good: −2° to 2°: 8.9 ± 3.9; Bad: 3° or 4°: 9.2 ± 2.5; Bad ≥ 5°: 9.3 ± 3.2.	Good alignment lead to better function with quicker rehabilitation, earlier hospital discharge, which could improve outcomes in TKA

Abbreviations: AP, anteroposterior; CT, computed tomography; FCP, femoral component position; FKSS, functional KSS; FS, functional score; KA, kinematic alignment; KS, knee score; LOS, length of stay; MA, mechanical alignment; PCA, posterior condylar axis; postop, postoperative; preop, preoperative; PTS, posterior tibial slope; ROM, range of motion; sFCA, sagittal femoral component angle; sTCA, sagittal tibial component angle; TCP, tibial component position; TFA, tibiofemoral angle; TKA, total knee arthroplasty; tKSS, total KSS.

**FIGURE 3:**
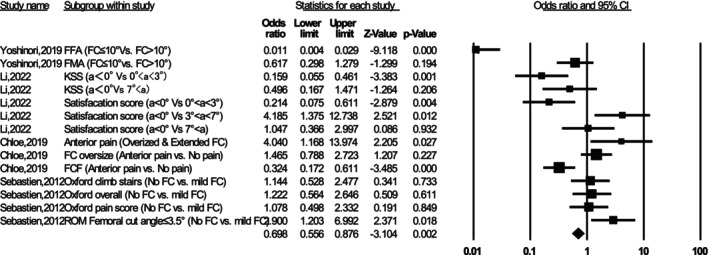
Forest plot comparing postoperative outcomes between proper sagittal alignment (Group A) and improper sagittal alignment (Group B) after primary TKA.

Optimized patient‐reported outcome measures (PROMs) correlated with native SA, whereas improper SA could lead to complications such as flexion contracture and patellar maltracking (Table S2). Okamoto et al. [15] found that the FCA of 7.3° ± 1.4° (OR = 3.73) was an independent predictor for flexion contracture > 10°. Lustig et al. [16] concluded that osteotomy of the distal femoral cut differing by > 3.5° from the MA increased the risk of contracture by 2.9 times. Li et al. [17] recommended that FCA between 0° and 3° might be a safe zone. Nakahara et al. [18] determined that sagittal cutting in the range of 3°–5° extension could increase the femoral anterior–posterior dimension by 2–3 mm. Additionally, two studies discussed ROM in relation to SA. Scott et al. [19] confirmed that patella baja is a predictor of anterior knee pain. Keshmiri et al. [20] observed that the patella shifted laterally between 30° and 60° flexion, with increased lateral tilt at 90° flexion. They concluded that restoring the preoperative sagittal profile and maintaining tibiofemoral balance is essential. Figure 3 presents a meta‐analysis comparing the risk of postoperative outcomes between two groups in primary TKA: Group A (proper sagittal alignment) and Group B (improper sagittal alignment). A fixed‐effects model was applied and the analysis showed a significant association between improper sagittal alignment and increased worsen outcomes (heterogeneity *I* ^2^ = 89.79%, pooled OR = 0.698, 95% CI: 0.556–0.876, *p* < 0.05). Notably, an OR < 1 indicates that improper alignment (Group B) is associated with a higher risk of worsen outcomes and complications relative to proper alignment (Group A). The egger's regression test showed no evidence of significant publication bias (*p* = 0.806).

### 3.4 Functional Outcomes and SA

Functional outcomes were also found to be associated with SA (Table 5). Guruprasad et al. [21] found that American Knee Society scores (AKSS) were significantly correlated with FCA (91°–95°) and TCA (86°–90°) (*p* < 0.001). Talmo et al. [22] noted that intramedullary instrumentation produced excellent tibial component slopes, with cruciate‐retaining (CR) implants achieving 3.89° ± 1.96° and posterior‐substituting (PS) implants achieving 1.7° ± 1.92°. Bar Ziv et al. [23] observed that patients with excessive tibial component slopes (> 5°; 66.7%) tended to achieve better minimal clinically important differences (MCID) in the Knee Injury and Osteoarthritis Outcome Score (KOOS) symptoms. Farooq et al. [24] found that posterior TS ≤ 4° and FCA ≤ 7° were correlated with superior PROMs. In another machine learning model, Farooq et al. [25] predicted patient satisfaction with changes closer to the native posterior TS (−2° to +2°) and FCA (0° to +7°) (*p* < 0.05).

**TABLE 5:** Sagittal alignments of components affect the prognosis.

Author, country (year)	Sample (knees) methords	Results 1	Results 2	Results 3	Results 4	Findings
Guruprasad et al., India (2023)	52 (81) patients X‐ray	Group TCA (86°–90°): AKSS increased to 62.13 ± 3.41 at 1 month from a preop of 32.94 ± 2.79, to 82.15 ± 2.74 and 87.03 ± 2.18 at the 6th month and 3rd year	Group FCA (91°–95°): AKSS increased to 63.00 ± 2.77 at 1 month from a preop of 33.45 ± 2.72, to 82.5 ± 2.74 and 87.75 ± 2.31 at the 6th month and 3rd year	Higher AKSS in group FCA in extension (91°–95°) and TCA (86°–90°)	Good correlation between sagittal alignments with AKSS	For the better functional outcome proper sagittal alignment of FC and TC in TKA is necessary
Talmo et al., USA (2010)	192 patients X‐ray	TC slope: CR vs. PS: 3.89° ± 1.96° (−0.6° to 10.3°) vs. 1.7° ± 1.92° (−2.9° to 6.7°)	The ideal TC slope is 0° for PS devices and 3° for CR devices	TC alignment: 90.00°; inliers: 99% ≤ 3°; outliers: 1% > 3°. Of a neutral tibial MA: 93% ≤ 2°; 70% ≤ 1°	The tibial component alignment was accurate with intramedullary tibial technique.	Tibial instrumentation resulted in excellent component slope and lower extremity alignment
Yaron et al., Israel (2022)	337 patients EOS	TC slpoe: Moderate vs. excessive: (0°–5°) vs. (PTS > 5°); 33.2% vs. 66.8%	Preop PTS: 9.6° ± 5.4° vs. 11.79° ± 5.4°. Postop PTS: 2.87° ± 1.8° vs. 9.19° ± 2.9°	Percentage of MCID in the KOOS symptoms: (67/112, 84%) vs. (113/225, 82%)	No differences in the average VAS, OKS, or the various subtypes of the KOOS score	Unrestricted KA and the excessive PTS seem to be reliable and safe
Farooq et al., USA (2023)	1311 patients X‐ray	Postop HKA and change of TC slpoe were predictive for achieving MCID in 90%	TC slpoe ≤ 4° and FF (−2° to 7°) correlated with MCID and superior PROM	Preop varus (valgus) and neutral knees likely to meet (MCID) and superior PROM	Implant alignments optimize PROMs, highlighting proper targets alignment	Sagittal and coronal alignment optimize PROMs, highlighting the importance of implant alignment targets
Farooq et al., Indiana (2021)	1091 patients X‐ray	Better outcome was predicted with a change of PTS (−2° to +2°) and FCF (0°–7°)	Worse outcomes were predicted with change of PTS > 5° and FCE&FCF > 10°	“Always” feel normal had a higher change in PTS (closer to 0 or native slope) than “never” feel normal knees	FCF and change of PTS did not influence pain with level walking or UCLA activity level scores	Superior PROM was predicted with native PTS and incorporating FCF

Abbreviations: AKSS, American Knee Society score; CR, cruciate‐retaining; FC, femoral component; FCA, femoral component angle; FCE, femoral component extension; FCF, femoral component flexion; HKA, hip–knee–ankle; IQR, interquartile range; KOOS, knee injury and osteoarthritis outcome score; MA, mechanical alignment; MCID, minimal clinically important differences; PROM, patient‐reported outcome measures; PS, posterior‐substituting; PTS, posterior tibial slope; TC, tibial component; TCA, tibial component angle.

### 3.5 Alignments of FBA and FCA

FBA also affects component alignments (Table S3). Seo et al. [26] calculated that the axis of the distal femoral anterior cortex (4.1° ± 2.8°, range 1.5°–11.7°) correlated with FBA (13.9° ± 4.2°, range 6.2°–24.5°) (*p* < 0.0001), implying that the palpable sagittal axis was associated with the sagittal mechanical axes, irrespective of the severity of the femoral sagittal axes. Zeng et al. [27] found gender differences in FBA (12.66° ± 1.98°), which correlated with the anterior distance of the proper entry point, suggesting that the position of the femoral entry point should be measured individually based on FBA. Zhang et al. [28] measured FBA (9.34° ± 3.56°, range 1°–16°) and found that it correlated with FCA (3.91° ± 3.15°, range −1° to −13°), influencing the sagittal femoral component alignment.

### 3.6 Variability of TS and the Need for Personalization in TKA (Table 6)

**TABLE 6:** Variation of PTS was observed in the sagittal plane.

Author, country (year)	Sample (knees) methords	Results 1	Results 2	Results 3	Results 4	Findings
Kaushik et al., Australia (2022)	4116 patients CT	mPTS: 8.9° ± 4.3°, (10.8%, 8°–9°); lPTS: 7.7° ± 4.3°, (11.2%, 7°–8°)	14.5% greater lPTS; 31.0% greater mPTS; 54.5% same slope ≤ 3°	HKA: −4° ± 5.5° (18° to −22°); (8.9%, −5° to −6°)	MPTA: −3.6° ± 3.0° (20° to −18°); LDFA: 3.0° ± 2.6° (−12° to 12°)	Variation of PTS and differential slope between the medial and lateral tibial plateau might result dissatisfaction with the existing techniques
Gokhan et al., USA (2015)	13,546 patients CT	PTS: 7.2° ± 3.7° (5° to −25°); 35.0% outliers, 11.6% < 4°, 19.1% > 10°	Males: 7.17° ± 3.82°, females: 7.24° ± 3.57°; short < 5′1″: 6.98° ± 3.94°; Tall > 6′0″: 6.98° ± 3.94°	Varus > 10°: 8.69° ± 5.29°; valgus ≥ 10°: 7° ± 4.12°; FBA > 5° (7.23° ± 4.18°); no FBA (7.17° ± 3.56°)	Africa: 5.77° ± 6.06°; Asia: 9.49° ± 4.52°; Australia: 6.14° ± 3.14°; Europe: 6.15° ± 3.78°	PTS is highly variable in osteoartic patients, which should be useful to determine optimum techniques and methodology to perform more accurate TKA
Douglas et al., USA (2017)	569 (1138) cadaverics 3‐D digitizer	mPTS: 7.3° ± 3.8°; lPTS: 5.7° ± 3.7°	Female and African‐American race have increased PTS	mPCO: 32.4 mm ± 3.9 mm; lPCO: 31.6 mm ± 4.0 mm	Decreased PCO were associated with increased PTS	Wide range of normal values in clinically, which provide valuable emphasis on sagittal plane sizing and positioning
Jae et al., Korea (2008)	66 (90) patients X‐ray	PTS: 10.6° ± 3.5° (1.9°–19.6°)	Angle between sMA: ACL: −3.2° ± 1.3°; PAA: −0.2° ± 1.0°; CAA: −2.2° ± 0.7°; PCL: 2.9° ± 1.1°; FSA: 1.1° ± 1.1°	PTS with regards to ACL 13.8° ± 3.5°; PAA 10.8° ± 3.4°; CAA 12.9° ± 3.8°; PCL 7.8° ± 3.5°; FSA 9.5° ± 3.9°	ACL (100%), PAA (60%), and CAA (100%) tilted anteriorly while PCL (100%) and FSA (86%) tilted posteriorly to the sMA	PTS varied widely among OA patients and varied depending on the references. The anatomical reference should be identified when PTS is used to evaluate the sagittal alignment of TKA
Hyuk et al., Korea (2008)	72 (133) patients 3D‐CT	mPTS: 8.7° ± 3.1°; lPTS: 7.4° ± 3.6°	Angles between sMA: TAA: 0.9° ± 0.67°; ATC: 2.2° ± 0.92°; FSA: −2.1° ± 0.98°	mPTS: MA: 8.7° ± 3.1°; TAA: 10° ± 3.3°; ATC: 12° ± 3.3°; FSA: 7.3° ± 3.2°	lPTS: MA: 7.4° ± 3.6°; TAA: 8.4° ± 3.8°; ATC: 9.6° ± 3.9°; FSA: 5.3° ± 4.0°	lPTS was different from mPTS; lPTS is better than mPTS respect to the restoration of natural PTS; PTS are dependent on the chosen reference axis and they were correlated with each other

Abbreviations: ACL, anterior cortical line; ATC, anterior tibial cortex; CAA, central anatomical axis; CT, computed tomography; FBA, femoral bowing angle; FSA, fibular shaft axis; HKA, hip–knee–ankle; LDFA, lateral distal femoral angle; lPCO, lateral posterior condylar offset; lPTS, lateral posterior tibial slope; mPCO, medial posterior condylar offset; MPTA, mechanical proximal tibial angle; mPTS, medial posterior tibial slope; PAA, proximal anatomical axis; PCL, posterior cortical line; PCO, posterior condylar offset; PTS, posterior tibial slope; sMA, sagittal mechanical axis; TKA, total knee arthroplasty.

**FIGURE 4:**
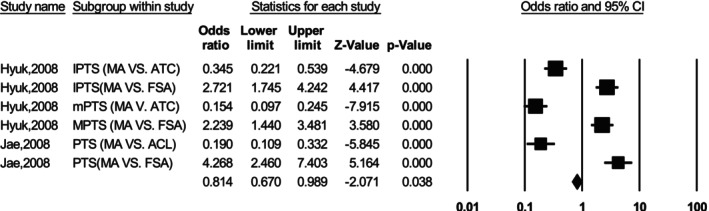
Forest plot comparing posterior tibial slope measurements using mechanical alignment (Group A) and alternative reference (Group B) in primary TKA.

Hazratwala et al. [29] found that the medial posterior TS and lateral posterior TS ranged from 5° anterior to 25°, with 22.6% of cases showing a differential posterior TS > 5°. Meric et al. [30] measured the posterior TS (7.2° ± 3.7°, range −5° to 25°; males: 7.17° ± 3.82°; females: 7.24° ± 3.57°) and found that 35.0% of cases were outliers, with 11.6% showing posterior TS < 4° and 19.1% showing posterior TS > 10°. Weinberg et al. [31] concluded that the medial posterior TS (7.3° ± 3.8°) was greater than the lateral posterior TS (5.7° ± 3.7°). These findings indicate significant variability in posterior TS, with correlations observed between different anatomical references. Han et al. [32] compared five anatomical references and found various relationships. However, Yoo et al. [33] concluded that the posterior TS relative to the sagittal MA was 10.6°. Several studies have examined the influence of different reference lines on the measurement of posterior TS in primary TKA. Figure 4 presents a meta‐analysis comparing two groups: Group A (measurements of posterior TS using a mechanical alignment line) and Group B (measurements of posterior TS using an alternative reference line). A fixed‐effects model was applied and the pooled analysis showed that the choice of reference line significantly affects the measured posterior TS, with pooled OR = 0.814 (95% CI: 0.670–0.989, *p* < 0.05) and substantial heterogeneity (*I* ^2^ = 97.12%). Egger's regression test showed no evidence of significant publication bias (*p* = 0.890).

### 3.7 Application of Novel Technologies to Achieve Optimal SA (Table 7)

**TABLE 7:** Novel technologies for optional sagittal alignments.

Author, country (year)	Sample (knees) methods	Results 1	Results 2	Results 3	Results 4	Findings
Takehito et al., Japan (2015)	11 (12) patients CT and X‐ray	Flexion gap: 19.1 ± 2.2 mm; extension gap: 12.3 ± 3.3 mm; gap difference: 6.8 ± 4.0 mm	Angle of average error made by PSI between preop and postop alignments ≤ 3°	Preop extension: −14.6° (−30° to 0°); flexion: 117.0° (90°–130°); TFA: 182.9° (178°–194°).	Preop sTFA flexion: 15.3°; postop sTFA flexion: 6.1°	Flexion gap is larger than the extension gap and should not aim at equal gaps in PS‐TKA
Bryce et al., USA (2023)	50 patients X‐ray	A DMR of 0.24 allowed accurate reproduction of the DMP and SMA.	Application of DMR to measure femoral flexion in the presence of femoral implants.	The DMR demonstrated angular variance between tSMA and cSMA < 1°	The DMR demonstrated linear distance between tDMP and cDMP < 1 mm	DMR is a valuable tool to evaluate sagittal alignments with landmarks absent
Tadashi et al., Japan (2016)	91 (100) patients CT	The distance required to make the guide rod parallel to the tibial sMA can be calculated.	Mean angle of deviation from the sMA: 0.9° ± 0.7° (0°–4.3°).	Increase 3.5 mm of the distance between the skin surface 20 cm below the resection plane results in 1° increase of PTS	The cutting guide parallel to the tibial sMA by making the distance equal to a product‐specific distance	The distance between the skin surface and the guide rod can be a useful guide for the PTS
Seo et al., Korea (2018)	222 (276) patients X‐ray	Of 17.4% outliers (alignment deviation > 2°) in the tibial sagittal plane	Outliers were associated with preop (OR 0.886) and postop (OR 0.803) flexion contractures	Correlations between bone resection surfaces and component alignment.	Difference between the medial and lateral gaps in extension ≥ 3 mm and poor bone quality were associated with outlier.	Awareness of factors related to alignment deviations and can help diminish the outliers
Solayar et al., China (2017)	58 patients CT and x‐ray	CT vs. x‐ray of SFA: −0.07° (−3.79° to 3.66°) and − 2.04° (−8.03° to 3.94°)	CT vs. x‐ray of STA: 0.21° (−2.76° to 3.2°) and−0.72° (−4.72° to 3.29°)	The correlation figures were lower for STA and worst for SFA	Anterior femoral bowing was significant in giving rise to poor observer correlation	Radiological remain a practical measurement for HKA and STA rather than SFA
Ryo et al., Japan (2012)	20 patients X‐ray	Of sagittal alignments: conventional vs. navigated: 3.2° ± 1.7° vs. 6.3° ± 2.0°	Different reference points on the distal femoral condyles for navigation systems resulted in differences up to 3.0° ± 1.5°	The insertion point of the intramedullary rod was preferred over the reference point on the distal femur	Navigation systems resulted in a more hyperextended position between the femoral and tibial components	Sagittal alignments achieved using the conventional technique and navigation systems is different
Chen et al., China (2014)	36 (72) patients X‐ray	Of sagittal TFA, conventional vs. navigated: (2.25° ± 3.14° vs. 1.19° ± 1.56°)	Of neutral alignment > 3°: conventional vs. navigated: 27.8% vs. 8.3%	Conventional vs. navigated: flexion (2.77° ± 2.21°) vs. hyperextension (−0.35° ± 1.45°)	No significant difference of KSS between the two groups	Navigated systems resulted in hyperextension of the FC than conventional technique

Abbreviations: cDMP, calculated distal mechanical point; cSMA, calculated sagittal mechanical axis; CT, computer tomography; DMP, distal mechanical point; DMR, distal mechanical ratio (DMR calculated as G/F; G is the distance from the distal anterior cortical axis to the sagittal mechanical axis, F is the distance from the distal anterior cortical axis to the posterior condylar cortex); FC, femoral components; HKA, hip–knee–ankle; KSS, knee society score; OR, odd ratio; postop, postoperative; preop, preoperative; PS, posterior cruciate ligament sacrificed; PSI, patient‐specific instrumentation; PTS, posterior tibial slope; SFA, sagittal femoral angle; sMA, sagittal mechanical alignments; STA, sagittal tibial angle; tDMP, true distal mechanical point; TFA, tibial femoral angle; TKA, total knee arthroplasty; tSMA, true sagittal mechanical axis.

**FIGURE 5:**
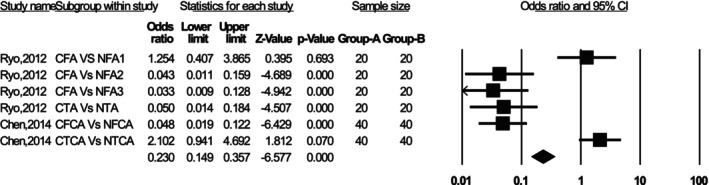
Forest plot comparing component alignment accuracy between conventional technique (Group A) and navigated technique (Group B) in primary TKA.

Kim et al. [34] found no significant differences between robotic‐assisted TKA and conventional TKA in terms of functional outcomes, aseptic loosening, overall survivorship, and complications. However, angular differences between the axes referenced by robotic‐assisted TKA and those used in conventional TKA were noted, with patient height potentially influencing these variations. Understanding these differences is crucial when evaluating implant positioning and surgical outcomes following robot‐assisted TKA. Additionally, caution is advised when assessing the flexion–extension angle of the knee, as the angles displayed in the Mako system differ from those measured using intramedullary anatomical axes. While SA principles vary between robotic‐assisted and conventional TKA, further studies are needed to determine which approach is most appropriate [35]. Figure 5 presents a meta‐analysis comparing the accuracy of component alignment between two surgical techniques in primary TKA: Group A (conventional technique) and Group B (navigated technique). The outcome of interest was the achievement of precise alignment. The pooled analysis showed that conventional surgery is significantly less accurate than navigated surgery in achieving precise alignment (heterogeneity *I* ^2^ = 92.673%, pooled OR = 0.230, 95% CI: 0.149–0.357, *p* < 0.001). An OR < 1 indicates that the conventional technique (Group A) is associated with lower accuracy compared to the navigated technique (Group B). Egger's regression test showed no evidence of significant publication bias (*p* = 0.179).

Hananouchi et al. [36] found that the flexion gap (19.1 mm) was larger than the extension gap (12.3 mm) when using patient‐specific instruments (PSI). Allen et al. [37] indicated that a distal mechanical ratio (DMR) of 0.24, as calculated in Table 7, reliably predicted a true sagittal MA ≤ 1° and a true distal mechanical point (DMP) ≤ 1 mm. Tsukeoka and Tsuneizumi [38] determined that the angle between the MA and a line parallel to the sagittal MA was 0.9° ± 0.7° (with 95% ≤ 2° and 99% ≤ 3°). Seo et al. [39] measured alignment deviation > 2°, which was associated with flexion contractures (preoperative OR 0.886 vs. postoperative OR 0.803). Solayar et al. [40] concluded that radiography might be less accurate than computed tomography (CT) in estimating prosthetic alignments. Sugama et al. [41] found differences of up to 3.0° ± 1.5° between navigation systems using different reference landmarks on the distal femoral condyle. Chen et al. [42] detected that femoral component alignments tended to show hyperextension with navigation (−0.35°) compared to conventional techniques (2.77°).

In contrast to coronal alignment, there has not been a unanimous definition of SA for either robotic‐assisted or manual TKA. In robotic‐assisted TKA, the femoral MA is defined by a line connecting the femoral head center to the Mako femoral knee center, while the tibial MA is defined by a line connecting the Mako tibial knee center to the Mako ankle center [43]. These differing definitions can lead to variations in clinical results. Femoral sagittal axis discrepancies may result in femoral cortical notching or prosthesis anterior flange overhanging, while tibial sagittal axis discrepancies may affect the posterior TS. Thus, SA obtained using conventional and navigation techniques differs. Based on available evidence, surgeons should aim for optimal SA with the assistance of novel technologies. A summary of comparisons of key outcomes is presented in Table 8.

**TABLE 8:** Summary of analysis in primary TKA for proper sagittal alignment.

Category	Parameters	Key findings
Significance of SA	PTS, ROM, HKA	Higher PTS correlates with reduced maximum flexion angle and flexion ROM; also correlates with posterior shift of medial compartment AP translation during step‐up and reduced lateral compartment AP translation during step‐down. Good three‐dimensional alignment improves rehabilitation, earlier discharge, and overall outcomes.
Component SA and prognosis	PTS, KA, FC, TC, PROMs, FCF	Proper sagittal alignment of femoral and tibial components (including native PTS and femoral component flexion) optimizes PROMs. Unrestricted KA and excessive PTS appear reliable and safe. Tibial instrumentation achieves excellent component slope and lower limb alignment.
Variability of PTS	PTS, lPTS, mPTS	PTS varies widely among patients and depends on the chosen reference axis. Lateral PTS differs from medial PTS, and lateral PTS is superior for restoring natural PTS. Anatomical references must be identified when using PTS to evaluate SA.
Novel technologies for optimal SA	PS‐TKA, HKA, SFA, FC	In PS‐TKA, flexion gap > extension gap; equal gaps should not be the goal. Navigated systems differ from conventional techniques in femoral component sagittal positioning (navigation more often results in femoral component hyperextension).
Complications of improper SA	Contracture, patella baja, pain	FCF > 3.5° increases risk of flexion contracture. Femoral osteotomy in ≥ 3° flexion or extension can lead to component sizing errors. FCF and patella baja predict anterior knee pain. Component alignment alters patellar kinematics.
Effect of FBA on SA	FBA, sFBA, cFBA, FCA	Palpable sagittal axis correlates with mechanical axis regardless of FBA. sFBA correlates with cFBA and affects FCA. Patients with sFBA often present with noncoronal femoral shaft bow.

Abbreviations: AP, anterior–posterior; cFBA, coronal femoral bowing angle; FBA, femoral bowing angle; FC, femoral component; FCA, femoral component angle; FCF, femoral component flexion; HKA, hip–knee–ankle angle; KA, kinematic alignment; lPTS, lateral posterior tibial slope; PTS, medial posterior tibial slope; OA, osteoarthritis; PROM, patient‐reported outcome measure; PS, posterior‐substituting; PTS, posterior tibial slope; ROM, range of motion; SA, sagittal alignment; SFA, sagittal femoral angle; sFBA, sagittal femoral bowing angle; TC, tibial component; TKA, total knee arthroplasty.

## 4 Discussion

### 4.1 Summary of Main Findings

TKA is a highly validated procedure known to yield favorable outcomes. However, SA has received comparatively less attention, and its functional implications remain controversial. This may be due to the challenges in radiographic measurements, where landmarks are not well‐defined and difficult to evaluate. In the sagittal plane balance, flexion contracture leads to restricted function, while excessive extension is associated with femoral notching [44, 45]. Despite considerable research, the optimal SA in TKA remains controversial. Therefore, it is essential to identify and validate the proper SA for TKA. The current study highlighted the variability in the recommendations due to differences in populations, surgical techniques, and outcome measures, providing a clearer understanding of the current evidence. Based on current reviews, the recommended safe zones are as follows: posterior TS is 0°–7°, TCA is 0°–5°, FCA is 0°–3°, FBA is 5°–10°, and TFA is 0° ± 3°. It should be noted that these values remain subject to individual patient anatomy and specific clinical scenarios; thus, slight deviations may be acceptable depending on the overall surgical plan.

### 4.2 Proper Sagittal Alignment Is Critical for Optimal TKA Outcomes

Surgeons should pay close attention to the SA of components, as it directly influences patellar kinematics. This consideration is crucial during surgery. Furthermore, navigated techniques such as the Mako system might present a higher risk of decreased posterior TS and hyperextension of the femoral prosthesis compared to manual TKA [43]. The variation in angles and measurements may be influenced by factors such as prosthesis design and manufacturer recommendations [46]. Evidence suggests that favorable functional outcomes are associated with proper SA. Prosthetic placement within 3° of normal alignment in all three planes has been linked to improved outcomes. Specifically, in the sagittal plane, positioning the femoral prosthesis with slight flexion (0°–3°) and the tibial prosthesis with a posterior TS (0°–7°) has been shown to enhance function [47]. Additionally, patients with lower cumulative error scores tend to experience quicker rehabilitation. The Figure 3 revealed adverse outcomes include restricted knee motion, decreased functional scores, anterior knee pain, reduced patient satisfaction, and even limitations in ROM, which suggest that sagittal alignment is a key factor influencing complications. We therefore chose to synthesize these studies together in order to underscore the serious clinical consequences of malalignment, which should be given sufficient attention in both research and practice.

Addressing sagittal malalignment is critical not only for preventing complications, but also for optimizing patient‐centered outcomes. Several studies have demonstrated that deviations beyond recommended alignment ranges are associated with lower PROMs, reduced satisfaction, and impaired functional recovery. Importantly, in some reports these decrements exceed the MCIDs, indicating that malalignment has a meaningful impact on patients' perceived benefit from surgery. These findings highlight the need for precise alignment to improve patient satisfaction, functional outcomes, and long‐term success of TKA.

### 4.3 Tibial Slope Measurement and Its Impact on TKA Outcomes

The TS impacts outcomes by affecting tibiofemoral joint contact pressure and balancing gaps. Inadequate slope has been identified as an independent risk factor for tightness during flexion under weight‐bearing conditions [48]. Flexion contracture may further illustrate the role of slope in maintaining balance and distributing load. The slope is influenced by the reference points used. Typically, accurate slope measurement can be achieved through a precise tibial cut within a cutting block. The distance between the skin surface and the rod serves as a valuable indicator. The anterior tibial crest, due to its palpability and minimal soft tissue coverage, is often used as a reliable reference. Excessive fat distribution in the lower limbs does not affect tibial component placement [49]. To accurately measure the sagittal reference, the distance from the tibial cut surface to the anterior skin should be 20 cm below in lateral radiographs [50]. However, surgeons must take caution to avoid rotational errors and ensure proper alignment of the cutting jig during osteotomy [51]. The presumed optimal methods, however, may not accommodate the diversity in anatomical structures across populations. The Figure 4 analyzed the variation of slope that obtained from different reference lines, and highlighted the importance for surgeons to use reliable and consistent reference lines in order to achieve precise posterior TS measurements and ensure reproducibility across studies. Variations in bi‐plateau disparity, patellofemoral joint kinematics, and morphological differences necessitate careful attention from surgeons.

### 4.4 Advanced Technologies Improve Sagittal Alignment Precision in TKA

Technological advancements have been introduced to achieve proper SA. Femoral cutting affects the accuracy of component positioning. Although the intramedullary guide is widely used, its effectiveness is compromised by inaccuracies arising from factors such as entry point, FBA, canal diameter, and rod structure [52]. A study demonstrated that pre‐operative CT scans improve postoperative alignment accuracy [53]. Therefore, individual measurement of the entry point, particularly, based on FBA, is recommended. To achieve optimal SA, both conventional TKA and navigation‐based TKA techniques vary in their ability to restore sagittal anatomy and balance gaps. Simultaneously, the application of navigation‐based and gap‐balanced techniques should be utilized [54]. Navigation systems tend to lead to hyperextension (1°–4°), while conventional techniques typically result in 1° of flexion [55]. Advanced technologies such as navigation and robotics have been shown to improve consistency by reducing alignment outliers. Figure 5 specifically compares the femoral and tibial axes in SA between the two techniques, suggesting that navigated techniques may offer greater accuracy than conventional methods. Robot‐assisted TKA provides the highest precision in SA through real‐time feedback and robotic control. Navigation systems improve accuracy but are generally less precise than robotic systems. PSI offers moderate improvement, but its accuracy depends on preoperative imaging and guide quality. In terms of SA precision, robot‐assisted TKA is superior, followed by navigation systems, while PSI is the least precise. However, cost, operative time, and resource availability should be considered when selecting the appropriate technology for clinical application. Surgeons should be aware of these differences in the sagittal plane to avoid complications.

### 4.5 ROM Is Influenced by Prosthesis Design

ROM is a key measure of TKA success, with 90° considered the minimum requirement for daily activities. Factors such as diagnosis, prosthesis design, and alignment significantly influence ROM. Antony et al. found that FCA and PCO were correlated with ROM, suggesting that increasing PCO and adjusting the FCA within therapeutic ranges can improve ROM [56]. Thickened PCO, often seen in high‐flex implants, allows for enhanced flexion. Moreover, spinopelvic alignment interacts with lower extremity kinematics, and corrections to lower limb alignment can impact overall body alignment. Surgeons should assess whole‐body alignment since lower limb alignment correction might alter spinopelvic alignment [57]. When pelvic parameters are altered due to knee flexion post‐TKA, improvements in standing flexion and reductions in flexion contracture can be observed [58].

In addition, the influences of implant design, surgical technique, and patient‐specific factors on sagittal alignment outcomes are multifactorial and should be emphasized.

#### 4.5.1 Implant Design

PS and CR implants differ fundamentally in their sagittal plane kinematics. PS designs incorporate a cam‐post mechanism that engages during knee flexion, actively inducing femoral rollback and maintaining posterior femoral translation, which typically results in more consistent sagittal alignment regardless of posterior cruciate ligament (PCL) condition. In contrast, CR implants rely entirely on the native PCL for femoral rollback; thus, outcomes are highly dependent on PCL tension, integrity, and balancing. A PCL that is too tight may restrict flexion and limit posterior femoral translation, whereas a lax or deficient PCL can lead to paradoxical anterior femoral translation (i.e., “femoral lift‐off”), adversely altering sagittal alignment.

#### 4.5.2 Surgical Technique

Among technical variables, TS preparation and femoral component positioning are, particularly, critical. Each 1° increase in posterior TS has been shown to increase posterior tibial translation by approximately 1–2 mm in flexion, thereby shifting the tibiofemoral contact point posteriorly and affecting overall sagittal alignment. Conversely, excessive slope (> 7°) may increase shear forces and risk of posterior tibial subsidence, while insufficient slope (< 3°) can limit flexion and cause anterior impingement. Regarding femoral component positioning, a flexion error of just 2°–3° in femoral component placement can alter the patellofemoral and tibiofemoral contact mechanics, leading to reduced flexion range, altered joint line position, and abnormal sagittal plane alignment. Small deviations in the anterior–posterior position of the femoral component also affect the patellar tendon moment arm, influencing extensor mechanism efficiency.

#### 4.5.3 Patient‐Specific Factors

Obesity (BMI ≥ 30 kg/m^2^) increases baseline mechanical loading across the knee joint by two to three times during activities such as stair climbing, which amplifies soft tissue demands and may accelerate malalignment if component positioning is not adjusted accordingly. Obesity also complicates ligament balancing due to thicker periarticular soft tissue envelopes. Additionally, ethnic variations in baseline osseous anatomy—such as TS (e.g., East Asian populations often have less posterior slope compared to Caucasian populations) or FBA—necessitate individualized adjustments. For example, a patient with native posterior TS > 10° may require slope reduction during tibial preparation to avoid excessive posterior tibial translation, whereas a patient with slope < 3° may benefit from slight slope augmentation to improve flexion. Failure to account for such anatomical variations can result in sagittal malalignment despite technically correct component placement by standard measurements.

Collectively, these factors underscore the importance of tailoring alignment strategies to implant choice, surgical precision, and patient anatomy to optimize functional outcomes. Each of these factors can independently or synergistically impact the positioning of knee components, making it critical for surgeons to consider these variables in preoperative planning to minimize alignment errors and improve patient outcomes.

The comparisons summarized in Table 8 suggest that variables may affect sagittal alignment accuracy, but their superiority remains inconclusive based on the current evidence.

However, several limitations exist in this study. First, the systematic review could not identify the mechanisms responsible for achieving precise SA. More sagittal variables exist beyond those described. Despite conducting a comprehensive search in four major English‐language databases, not all relevant studies may have been included. Second, significant heterogeneity was observed in the sagittal alignment and functional outcome measures presented in figures. Upon reviewing the corresponding literature, we attribute this to differing research focuses and the lack of consensus regarding the optimal sagittal alignment. Therefore, while this heterogeneity warrants attention, it also offers valuable insights. Readers are advised to interpret the findings selectively based on their specific objectives. Due to the limited number of included studies, meaningful sensitivity analyses were precluded; however, the primary results based on the fixed‐effect model were relatively conservative, and the results demonstrated that the overall findings were robust and consistent. Furthermore, the scarcity of information emphasizes the need for further studies to validate the findings with supplementary data. Finally, it is essential to recognize that prolonged follow‐up studies are necessary to confirm the effectiveness of the current findings.

## 5 Conclusion

The optimal SA for TKA remains uncertain; however, it is hypothesized that achieving favorable SA could contribute to improved prognosis and enhanced functional outcomes. As discussions around SA continue, biomechanical, anatomical, and soft tissue considerations have been raised as important factors in achieving optimal surgical outcomes. We emphasize the importance of individualized patient care, considering factors such as implant design and patient characteristics, and also provide practical guidance on how these factors should be incorporated into surgical planning to optimize sagittal alignment outcomes. Target alignment values based on the latest evidence to achieve these goals using advanced technologies such as navigation systems and robotic‐assisted surgery. Understanding and implementing precise SA, tailored to individual patient needs, is crucial for enhancing the long‐term success of TKA procedures.

## Author Contributions

**Guoqing Li:** investigation, methodology, writing – original draft, writing – review and editing. **Yong Huang:** writing – review and editing. **Wang Deng:** writing – review and editing. **Ji Zhang:** writing – review and editing.

## Funding

The institution has received funding from the National Natural Science Foundation of China (No. 82402789), Beijing Jishuitan Hospital Youcai Plan (JSTYC202402), and Beijing Jishuitan Research Funding (No. HL202402), Beijing Natural Science Foundation (No. L232062, No. L222063).

## Ethics Statement

The authors have nothing to report.

## Consent

The authors have nothing to report.

## Conflicts of Interest

The authors declare no conflicts of interest.

## Supporting information


**Table S1:** Search strategies in per database.

**Table S2:** The complications of improper sagittal alignments after TKA.

**Table S3:** Femoral bowing angle affects the component alignment in the sagittal plane.

## Data Availability

Data are available from the corresponding authors upon reasonable request with the permission of the Department of Orthopedics in Beijing Jishuitan Hospital.
