# Supplementary material for: What Are the Optimal Sagittal Alignments in Primary Total Knee Arthroplasty: A Systematic Review and Meta‐Analysis
Source: Orthop Surg. 2026 May 12;18(6):1155–72. doi: 10.1111/os.70329 (PMC13238853; doi:10.1111/os.70329)
Supplement: Supplementary file 1 — Table S1: Search strategies in per database. [file OS-18-1155-s001.docx]

| **Supplementary TABLE 1** Search strategies in per database | | |
| --- | --- | --- |
| Database | Search Strategy | Number |
| Pubmed (MEDLINE) | (((sagittal[Title/Abstract]) OR (sagittal plane[Title/Abstract])) OR (sagittal vertical axis[Title/Abstract]))AND ((alignment[Title/Abstract]) OR (balance[Title/Abstract]))) AND ((((((((Arthroplasty, Replacement, Knee[MeSH Terms]) OR (Arthroplasty, Replacement, Knee[Title/Abstract])) OR (Total Knee Arthroplasty[Title/Abstract])) OR (Knee Replacement Arthroplasty[Title/Abstract])) OR (Unicompartmental Knee Arthroplasty[Title/Abstract])) OR (Unicondylar Knee Arthroplasty[Title/Abstract])) OR (Partial Knee Arthroplasty[Title/Abstract])) | 569 |
| the Cochrane library | ((sagittal):ti,ab,kw OR (sagittal plane):ti,ab,kw OR (sagittal vertical axis):ti,ab,kw) AND ((alignment):ti,ab,kw OR (balance):ti,ab,kw) AND ((Arthroplasty, Replacement, Knee):ti,ab,kw OR (Total Knee Arthroplasty):ti,ab,kw OR (Knee Replacement Arthroplasty):ti,ab,kw OR (Unicompartmental Knee Arthroplasty):ti,ab,kw OR (Unicondylar Knee Arthroplasty OR Partial Knee Arthroplasty):ti,ab,kw) | 100 |
| Embase | (sagittal:ti,ab OR (sagittal:ti,ab AND plane:ti,ab) OR (sagittal:ti,ab AND vertical:ti,ab AND axis:ti,ab)) AND (alignment:ti,ab OR balance:ti,ab) AND ((((((arthroplasty, AND replacement, AND knee:ti,ab OR total) AND knee AND arthroplasty:ti,ab OR knee) AND replacement AND arthroplasty:ti,ab OR unicompartmental) AND knee AND arthroplasty:ti,ab OR unicondylar) AND knee AND arthroplasty:ti,ab OR partial) AND knee AND arthroplasty) | 204 |
| Web of Science | "sagittal (Abstract) OR sagittal plane (Abstract) OR sagittal vertical axis (Abstract) AND "alignment (Abstract) OR balance (Abstract) and Preprint Citation Index "" AND ""Arthroplasty, Replacement, Knee (Abstract) OR Total Knee Arthroplasty (Abstract) OR Knee Replacement Arthroplasty (Abstract) OR Unicompartmental Knee Arthroplasty (Abstract) OR Unicondylar Knee Arthroplasty (Abstract) OR Partial Knee Arthroplasty (Abstract) """ | 524 |
