# Supplementary material for: What Are the Optimal Sagittal Alignments in Primary Total Knee Arthroplasty: A Systematic Review and Meta‐Analysis
Source: Orthop Surg. 2026 May 12;18(6):1155–72. doi: 10.1111/os.70329 (PMC13238853; doi:10.1111/os.70329)
Supplement: Supplementary file 2 — Table S2: The complications of improper sagittal alignments after TKA. [file OS-18-1155-s002.docx]

| **Supplementary TABLE 2** The complications of improper sagittal alignments after TKA | | | | | | |
| --- | --- | --- | --- | --- | --- | --- |
| Author  Country (Year) | Sample (knees) methords | Results 1 | Results 2 | Results 3 | Results 4 | Findings |
| Yoshinori et.al Japan (2019) | 106 (143) patients X-ray | 28% contracture >10° with FCA: 7.3°±1.4°;  72% contracture of ≤10° with FCA: 4.2°±1.2°. | The PTS did not correlate with either the extension or ﬂexion angle. | There was no difference with regard to pre- and post-op HKA. | FCA (OR=3.73) and height (OR=0.43) were retained as predictive variables for flexion contracture>10°. | The position of the femoral component in short patients may increase the risk of knee flexion contracture. |
| Sebastien et.al Australia (2012) | 95 patients | (flexion contracture≤5°) vs. (flexion contracture>5°):  66.32% vs. 33.68%. | The distal femoral cut orientation was different (3.15° vs. 3.77°). | Distal femoral cut angle>3.5° increased the risk of flexion contracture by 2.9-fold. | Post-op OKS was comparable among the self-reported pain, climb stairs, or overall score. | The FC should be implanted≤3.5° to reduce the risk of flexion contracture. |
| Li et.al China (2022) | 66 (87) patients X-ray | The α angle was associated with post-op knee extension. | α angle with best post-op KSS (0.57°), satisfaction (0.96°), ROM (-1.42°), lowest pelvic incidence (0.33°). | Better post-op KSS and higher satisfaction score with α angle from 0° to 3°. | The sagittal alignment of FC with increased pelvic incidence should be emphasized. | Extreme α angle was associated with poorer outcomes and 0 to 3° might be the acceptable range. |
| Hiroyuki Japan (2012) | 100 patients  3-D imaging | The AP dimension increased in 3° vs. 5° extension: 1.8 ± 0.6 mm (0.1 to 3.2 mm) vs. 3.1 ± 0.7 mm (0.7 to 5.2 mm). | The AP dimension decreased in 3° vs. 5° flexion: 1.5 ± 0.6 mm (0.1 to 3.5 mm) vs. 2.6 ± 0.6 mm (1.2 to 4.2 mm). | The AP dimension changed>2 mm (43%, 3°) and (96%, 5°) in extension while >2 mm (18%, 3°) and (83%, 5°) in flexion. | The change rates of FC size increased 2% (3°) and 57% (5°) in extension while decreased 2% (3°) and 31% (5°) in flexion. | Upsizing or downsizing of the FC can occur if the femoral osteotomy is performed in at least 3° ﬂexion or extension. |
| Chloe et.al Scotland (2019) | 297 patients X-ray | Mean VAS of 34.3 ± 25.1 (5 to 100) of 45.06% patients with anterior knee pain. | Pain vs. no pain:  FCA: -0.6° (21.5 to 0.3) vs. 1.42° (0.9 to 2.0);  AFO ratio: 17.2% (15.6 to 18.8) vs. 13.3% (11.1 to 15.5). | Flush femoral components were more ﬂexed (1.77°± 2.4°, 25°to 7°) than those that were not ﬂush (20.8°± 3.0°; -15°to 8°). | FCA was correlated with a reduced AFO ratio and an increased PCO ratio. | Femoral component flexion and ﬂush, patella baja could predict anterior knee pain in TKA patients. |
| Armin et.al Germany (2016) | 10 cadaveric X-ray | Both components alignment changed patellar mediolateral shift. | Change of FCA (0° to 5°) with 1° increase TC slope (4° to 6.5°) leads to a patellar lateral shift 17.3 mm. | An increase in FCA and TC slope decreased epicondylar distance and increased patella internal rotation. | An increase in FCA led to an increase in patellar lateral tilt, while higher TC slope led to a decrease in patellar lateral tilt. | Component alignment alters patellar kinematics, which should be considered during TKA. |
| Notes: femoral component angle, FCA; posterior tibial slpoe, PTS; hip-knee-ankle, HKA; odds ratio, OR; Oxford knee scores, OKS; α angle: the angle between the distal femur anterior cortex line and ﬂange of the femoral component; knee society score, KSS; range of motion, ROM; femoral component, FC; postoperative, post-op; anteroposterior, AP; visual analog scale, VAS; anterior femoral offset, AFO; medial proximal tibial angle, MPTA; posterior condylar offset, PCO; tibial component, TC; total knee arthroplasty, TKA. | | | | | | |
