# Supplementary material for: What Are the Optimal Sagittal Alignments in Primary Total Knee Arthroplasty: A Systematic Review and Meta‐Analysis
Source: Orthop Surg. 2026 May 12;18(6):1155–72. doi: 10.1111/os.70329 (PMC13238853; doi:10.1111/os.70329)
Supplement: Supplementary file 3 — Table S3: Femoral bowing angle affects the component alignment in the sagittal plane. [file OS-18-1155-s003.docx]

| **Supplementary TABLE 3** Femoral bowing angle affects the component alignment in the sagittal plane | | | | | | |
| --- | --- | --- | --- | --- | --- | --- |
| Author Country (Year) | Sample (knees) methords | Results 1 | Results 2 | Results 3 | Results 4 | Findings |
| Zeng et.al China (2022) | 80 Chinese CT | sFBA: 12.66° ± 1.98° (7.66° to 18.11°);  Male vs. female: 11.89°±1.66° vs. 13.42±2.00°. | Males: 0.17±1.86 mm (-4.13 to 4.77 mm) medial & 12.37 ± 2.39 mm (7.14 to 18.96 mm) anterior to the AIN. | Females: 0.02 ± 2.00 mm (-3.91 to 4.38 mm) medial & 16.13±2.72 mm (9.94 to 21.15 mm) anterior to the AIN. | Every 1° increase in the sFBA, the approach shifted anteriorly by an average of 1.15 mm. | Correlation between the entry point and the sFBA could direct sagittal alignment. |
| Seo et.al Korea (2009) | 76 patients X-ray | sFBA: 13.9°±4.2° (6.2° to 24.5°). | Distal femoral anterior cortex (4.1°±2.8°, 1.5° to 11.7°) was more flexed than the sMA. | Palpable sagittal axis (2.4°±0.9°, 0.4° to 4.2°) more flexed than the sMA. | The distal femoral anterior axis rather than palpable sagittal axis was correlated with FBA. | Palpable sagittal axis showed relationship with the sMA regardless of the of FBA. |
| Zhang et.al China (2021) | 71 (77) patients X-ray | sFBA: 9.34°±3.56° (1° to 16°); cFBA: 3.25°±3.79° (-7° to -17°); mTFA: 0.60°±1.95° (-3° to -6°); FCA: 3.91°±3.15° (-1° to -13°). | The sFBA was correlated with cFBA and FCA while the cFBA was correlated with mTFA. | Significant difference of FCA between the patients with sFSB and without sFSB. | Significant difference of mTFA between the patients with cFSB and without cFSB. | The sFBA was correlated with cFBA; the sFSB affect the FCA; the patients with sFSB usually presented non-cFSB. |
| Notes: Computed tomography, CT; sagittal femoral bowing angle, sFBA; intercondylar notch, AIN; sagittal mechanical axis, sMA; femoral bowing angle, FBA; femoral component angle, FCA; coronal femoral shaft bowing, cFSB; mechanical tibiofemoral angle, mTFA; sagittal femoral shaft bowing, sFSB. | | | | | | |
